# Intratumoral Distribution and pH-Dependent Drug Release of High Molecular Weight HPMA Copolymer Drug Conjugates Strongly Depend on Specific Tumor Substructure and Microenvironment

**DOI:** 10.3390/ijms21176029

**Published:** 2020-08-21

**Authors:** Anne-Kathrin Noack, Henrike Lucas, Petr Chytil, Tomáš Etrych, Karsten Mäder, Thomas Mueller

**Affiliations:** 1 Institute of Pharmacy, Martin Luther University Halle-Wittenberg, 06120 Halle (Saale), Germany; a-k.noack@gmx.net (A.-K.N.); henrike.lucas@pharmazie.uni-halle.de (H.L.); Karsten.Maeder@pharmazie.uni-halle.de (K.M.); 2 Institute of Macromolecular Chemistry, Czech Academy of Sciences, Heyrovský Sq. 2, 162 06 Prague 6, Czech Republic; chytil@imc.cas.cz (P.C.); etrych@imc.cas.cz (T.E.); 3 University Clinic for Internal Medicine IV, Hematology/Oncology, Medical Faculty of Martin Luther University Halle-Wittenberg, 06120 Halle (Saale), Germany

**Keywords:** HPMA copolymer, polymer drug conjugates, pH-sensitive drug release, tumor microenvironment, chemotherapy resistance

## Abstract

Stimulus-sensitive polymer drug conjugates based on high molecular weight N-(2-hydroxypropyl) methacrylamide (HPMA) copolymers carrying doxorubicin via a pH-dependent cleavable bond (pHPMA-Dox) were previously shown to be able to overcome multi-drug resistance. Nevertheless, a tumor type dependent differential response was observed. Although an improved and more selective tumor accumulation of pHPMA-Dox is generally achieved due to the enhanced permeability and retention (EPR) effect, little is known about the fate of these conjugates upon entering the tumor tissue, which could explain the different responses. In this study, we compared in vitro and in vivo accumulation and Dox-activation of pHPMA-Dox in three cancer cell line models (1411HP, A2780cis, HT29) and derived xenograft tumors using a near-infrared fluorescence-labeled pHPMA-Dox conjugate. Firstly, cytotoxicity assays using different pH conditions proved a stepwise, pH-dependent increase in cytotoxic activity and revealed comparable sensitivity among the cell lines. Using multispectral fluorescence microscopy, we were able to track the distribution of drug and polymeric carrier simultaneously on cellular and histological levels. Microscopic analyses of cell monolayers confirmed the assumed mechanism of cell internalization of the whole conjugate followed by intracellular cleavage and nuclear accumulation of Dox in all three cell lines. In contrast, intratumoral distribution and drug release in xenograft tumors were completely different and were associated with different tissue substructures and microenvironments analyzed by Azan- and Hypoxisense^®^-staining. In 1411HP tumors, large vessels and less hypoxic/acidic microenvironments were associated with a pattern resulting from consistent tissue distribution and cellular uptake as whole conjugate followed by intracellular drug release. In A2780cis tumors, an inconsistent pattern of distribution partly resulting from premature drug release was associated with a more hypoxic/acidic microenvironment, compacted tumor tissue with compressed vessels and specific pre-damaged tissue structures. A completely different distribution pattern was observed in HT29 tumors, resulting from high accumulation of polymer in abundant fibrotic structures, with small embedded vessels featuring this tumor type together with pronounced premature drug release due to the strongly hypoxic/acidic microenvironment. In conclusion, the pattern of intratumoral distribution and drug release strongly depends on the tumor substructure and microenvironment and may result in different degrees of therapeutic efficacy. This reflects the pronounced heterogeneity observed in the clinical application of nanomedicines and can be exploited for the future design of such conjugates.

## 1. Introduction

Nanoscaled drug delivery systems are useful tools to improve cancer therapy. They are designed to overcome the shortcomings of conventional chemotherapy, i.e., low tumor specificity, systemic toxicity and occurrence of resistance, by improving the pharmacokinetic profile and the therapeutic index of chemotherapeutic agents. The conception relies on the enhanced permeability and retention (EPR) effect, which mediates an increased accumulation of macromolecules preferentially in the tumor tissue due to the leaky tumor vasculature and the lack of functional lymphatic vessels [1]. In recent years, it has been increasingly recognized that this fundamental principle, which was established in preclinical models, cannot be directly translated to human tumors and the relevance of the EPR effect in the clinical treatment is controversially discussed [2,3,4,5,6]. Actually, there is large inter- and intra-individual heterogeneity explaining the heterogeneous outcomes of clinical trials, and several new strategies to improve EPR-mediated tumor targeting are under investigation [3,7]. In this regard, one important aspect is the process of intratumoral distribution of nano-carriers, which can contribute to this heterogeneity [8]. Our present study investigates the association between tumor type specific distribution pattern, stimulus dependent prodrug activation and treatment efficacy.

N-(2-hydroxypropyl) methacrylamide (HPMA) copolymer drug conjugates are widely studied tools in nanomedicine [9,10,11,12]. In this system, the cytotoxic drug is covalently bound to the polymeric carrier via a biodegradable linker, which enables controlled release of the active drug to achieve a pharmacological effect. There are several possibilities to achieve stimuli-responsive and thereby tumor site specific drug release such as by the usage of pH-sensitive, reduction-sensitive or enzymatic-cleavable linkers [9]. The combined mechanisms of EPR-mediated tumor targeting and tumor specific drug release may result in a more selective and effective tumor therapy.

In our recent study, we investigated a 200kDa high molecular weight star-like structured HPMA-copolymer drug conjugate with doxorubicin (Dox) linked via a pH-sensitive hydrazone bond (pHPMA-Dox) [13]. High therapeutic efficacy and the ability to overcome drug resistance with complete tumor regressions could be demonstrated in two xenograft tumor models (1411HP germ cell tumor, A2780cis ovarian carcinoma). The superior tumor response was associated with substantially higher intratumoral drug accumulation mediated by the conjugate after application of higher total doses compared to free Dox. Thus, the supposed mechanism mentioned above had worked in principle. However, the two models showed differences in the pattern of response. While the 1411HP tumors initially showed no response to the treatment followed by a delayed but strong response, the A2780cis tumors exhibited tumor growth inhibition immediately after the start of treatment. In addition, mice bearing A2780cis tumors showed earlier signs of toxicity and tolerated the therapy regimen somewhat less than 1411HP tumor mice. Investigations of the tumor microenvironment then revealed a more hypoxic/acidic tumor micromilieu in A2780cis tumors compared to 1411HP tumors, explaining the earlier response. In 1411HP tumors, a delayed, possibly therapy related switch to a more hypoxic/acidic environment could be observed, which was accompanied by the onset of response. Furthermore, it was postulated that a partly premature release of Dox, immediately after delivery of the conjugate in the tumor tissue, may lead to re-circulation of a part of Dox contributing to the higher toxicity observed in the A2780cis tumor model [13]. Further testing of the pHPMA-Dox conjugate in a third xenograft tumor model (HT29 colorectal carcinoma) revealed a completely different response pattern and failed to show tumor regression (see description in Section 2.1.). The occurrence of substantial toxicity restricted the application of sufficiently higher total doses, which explained, at least in part, the failed superior impact of pHPMA-Dox over free Dox observed in this model. Based on these findings, we hypothesized that the mechanism of accumulation and drug release of such conjugates can clearly differ depending on the tumor type and that the tumor response is influenced by the specific tumor substructure and microenvironment.

In preclinical mouse model systems, the clearly increased and more selective tumor accumulation of polymeric drug conjugates due to the EPR effect is generally proven. However, little is known about the fate of these conjugates upon entering the tumor tissue, which could explain the different responses. The general idea about the mechanism of cellular accumulation and drug release is based on in vitro investigations of cell monolayers using pHPMA conjugates with an enzymatically cleavable linker. From these studies, it is known that polymeric conjugates can enter the cells by multiple endocytic pathways depending on the physicochemical characteristics of the polymer carrier. They are mainly confined to the lysosomal compartment where the drug is released, but they are also able to enter the cytoplasm and can accumulate in the nucleus [14,15]. To enlighten the mechanisms of intratumoral distribution and drug release of polymeric drug conjugates, it is necessary to visualize both components simultaneously in real tumor tissues on a histological level. In our previous studies, we used fluorescence-labeled (DY-782) pHPMA-conjugates and multispectral fluorescence imaging to analyze tumor accumulation of polymer carrier and Dox or a fluorescent model drug, which was feasible for in vivo or ex vivo analysis due to accumulating signals derived from whole tissues [13,16,17]. However, it was not possible to detect the polymer carrier on a histological level on slides from fixed tumors, which hampered further mechanistic studies. Furthermore, the Dox-derived fluorescence signal could not be distinguished from the background due to the substantial autofluorescence of the tissue slides upon excitation with blue or green fluorescent light. For the current study, a new variant of the star-like structured pHPMA-Dox conjugate was synthesized, which was provided with a higher degree of labeling and used a more intensive, and fixing procedure resistant, far-red fluorescent label (Cy7). In addition, new equipment allowing multispectral fluorescence microscopy enabled spectrum-dependent isolation and clear allocation of signals, thereby separating the background fluorescence. Together, this enabled simultaneous tracking of Dox and polymer backbone to analyze intratumoral distribution and drug release of the conjugate on a histological level.

## 2. Results

### 2.1. Testing of the pHPMA-Dox Conjugate in the HT29 Colorectal Carcinoma Xenograft Model

**Figure 1:**
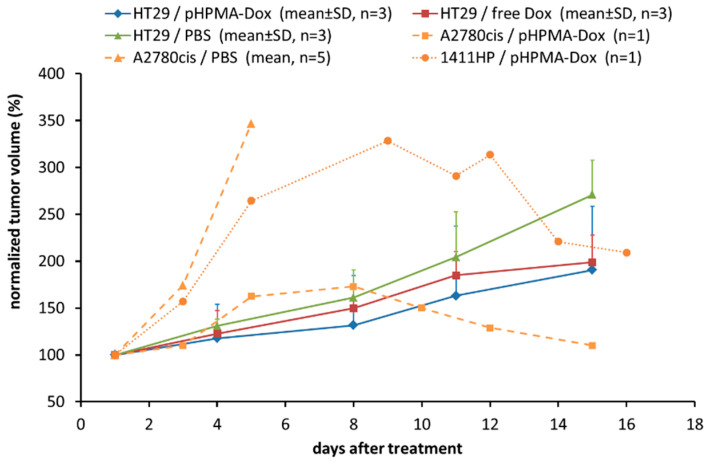
Tumor growth and treatment response pattern of HT29 tumors compared to 1411HP and A2780cis tumors. Mice were treated on days 1 and 4 with PBS or free Dox (5 mg/kg) or a 2-fold Dox equivalent dose of pHPMA-Dox. Depicted are the tumor volumes normalized to day 1 (start of treatment). The three graphs displaying 1411HP and A2780cis tumors are data of our previous study [13] and were reused here for comparison of the tumor models. The PBS graph of A2780cis is representative for growth of both A2780cis and 1411HP control tumors.

Further testing of the previously studied pHPMA-Dox conjugate in the HT29 colorectal carcinoma model failed to show clear superiority over free Dox, although this was clearly demonstrated for the 1411HP/A2780cis models [13]. As shown in Figure 1, treatment with free Dox and pHPMA-Dox resulted in similar response patterns, even though the latter inhibited tumor growth somewhat more. The pHPMA-Dox was administered as 2-fold doses equivalent to free Dox on days 1 and 4, in accordance with the scheme used in our previous study. However, no tumor regression could be achieved. Mice of both groups developed similar toxicity patterns and a third application was omitted. Figure 1 also displays selected graphs of our previous study to demonstrate the differences between the three xenograft tumor models. In the 1411HP/A2780cis models, application of two 2-fold doses of pHPMA-Dox did induce tumor regression after different extended lag phases (Figure 1). However, as recently shown, complete tumor regression could only be achieved by application of three 2-fold doses in both models; otherwise, tumor regrowth was observed after day 18 (not shown). Although 1411HP and A2780cis tumors clearly differ in their timing of response upon treatment with pHPMA-Dox, they also have similar characteristics when compared to HT29 tumors. Both show fast tumor growth and tumor regression after pHPMA-Dox treatment. Notably, in both tumor types, the response pattern after treatment with pHPMA-Dox compared to free Dox is completely different. In contrast, HT29 tumors exhibit rather slow growth and the response patterns upon treatment with free Dox and pHPMA-Dox are quite similar (Figure 1). Based on these findings, we supposed a tumor type specific, different mechanism of accumulation and drug release in the three tumor models.

### 2.2. Synthesis and Physico-Chemical Characterization of Cy7-Labeled Variant of the pHPMA-Dox Conjugate

**Scheme 1:**
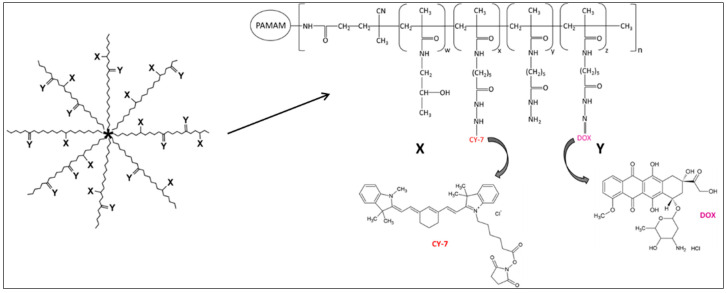
Structure of the pHPMA-Dox-Cy7 conjugate.

Investigating the mechanism of intratumoral distribution and drug release of the pHPMA-Dox conjugate requires a simultaneous visualization of both Dox and the polymer carrier. For this purpose, a new variant of the star-like structured pHPMA-Dox conjugate was synthesized comprising a similar amount of Dox bound via pH-sensitive linkage and an intensive far-red fluorescent label (Cy7) bound via biologically stable covalent hydrazide bond to the polymer carrier (Scheme 1). In addition, a higher degree of labeling could be achieved as compared with our previously used conjugate.

The star polymer was prepared by the controlled “grafting to” approach, which enabled the attachment of seven linear polymer chains to a small dendrimer core (Table 1). The grafting of linear polymers led to the significant increase in the molecular weight and size of the star polymer. While the molecular weight was increased approximately seven times, the hydrodynamic radius increased approximately three times. The increase in the hydrodynamic size enabled the star polymer to circulate for a longer time in the body, as the size exceeded the limit of the renal threshold and the polymer was removed from the organism by a slower process via the hepatobiliary way [17]. Prior to use, the star polymer conjugate was carefully freed of all impurities, unbound Dox and dye, using the gel chromatography in organic solvent.

### 2.3. Analysis of pH-Dependent Drug Relaease and Cellular Uptake of the pHPMA-Dox-Cy7 Conjugate

**Figure 2:**
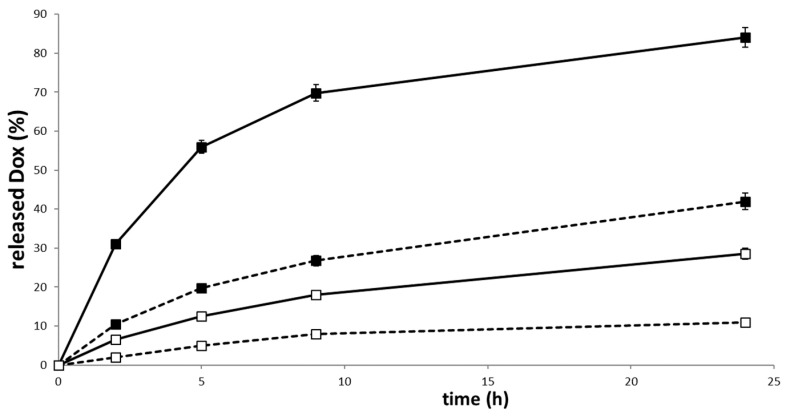
Release of free Dox from polymer conjugate at different pH levels at 37 °C for the indicated time periods. (■──) pH 5.0; (■- - -) pH 6.0; (□──) pH 6.5; (□- - -), pH 7.4.

First, we validated the functionality of pHPMA-Dox-Cy7 in terms of pH-dependent drug release. The polymer was incubated at various pH levels mimicking the blood stream (pH 7.4), extracellular space of the tumor (pH 6.5), endosomes (pH 6.0) and lysosomes (pH 5.0). Strong pH sensitivity was observed, showing the stability at neutral pH and rapid release at acidic pH of lysosomes (Figure 2). Even in the milieu of the extracellular space of the tumor, the drug is released more rapidly than in the bloodstream. The data clearly prove the applicability of the star polymer-Dox-Cy7 conjugate as an efficient pH-responsive delivery vehicle.

**Figure 3:**
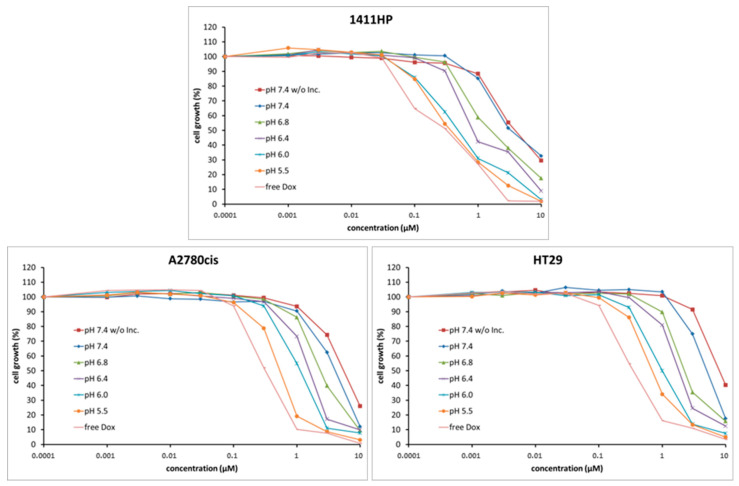
Analysis of pH-dependent drug release and activation of pHPMA-Dox-Cy7. The conjugate was pre-incubated in buffers with different pH values for 24 h and immediately used for the assay. The cell lines were treated for 2 h and analyzed after further incubation for 96 h in drug-free medium.

Second, we proved the functionality of pHPMA-Dox-Cy7 in terms of pH-dependent drug activation as well as cellular response to treatment using the three cell lines. To this end, the conjugate was pre-incubated in buffers with different pH values in a range of 5.5 to 7.4 for 24 h. Afterwards, it was used to prepare serial dilutions, which were directly used for the cytotoxicity assays in comparison to free Dox. A short treatment time of 2h was chosen, with the aim of preferentially capturing the impact of released Dox. As shown in Figure 3, a gradual lowering of the pH resulted in a stepwise increase in cytotoxicity, which is in clear accordance with the characteristics of our previously investigated conjugates. The pattern of pH-dependent increase in cytotoxicity was similar among all three cell lines. Based on IC_50_ values, a roughly ten-fold increase in cytotoxic activity between pH 7.4 and pH 5.5 was observable in each cell line. A comparison of the graphs for pH 7.4 with or without pre-incubation indicated the required stability of the conjugate at neutral conditions. After pre-incubation at pH 5.5, most of the Dox seemed to be released. Furthermore, the cell lines had very similar IC_50_ values of free Dox, which also confirmed the similar intrinsic drug resistance of HT29 cells as in 1411HP and A2780cis cells. In addition, a similar response to the uncleaved conjugate, which is represented by the pH 7.4 graphs, was observed, although 1411HP cells appeared to be somewhat more vulnerable, suggesting an accelerated uptake of the conjugate in this cell type. These results confirmed the mechanism of pH-dependent drug release of the pHPMA-Dox-Cy7 conjugate and revealed comparable responses of the three different cell lines.

**Figure 4:**
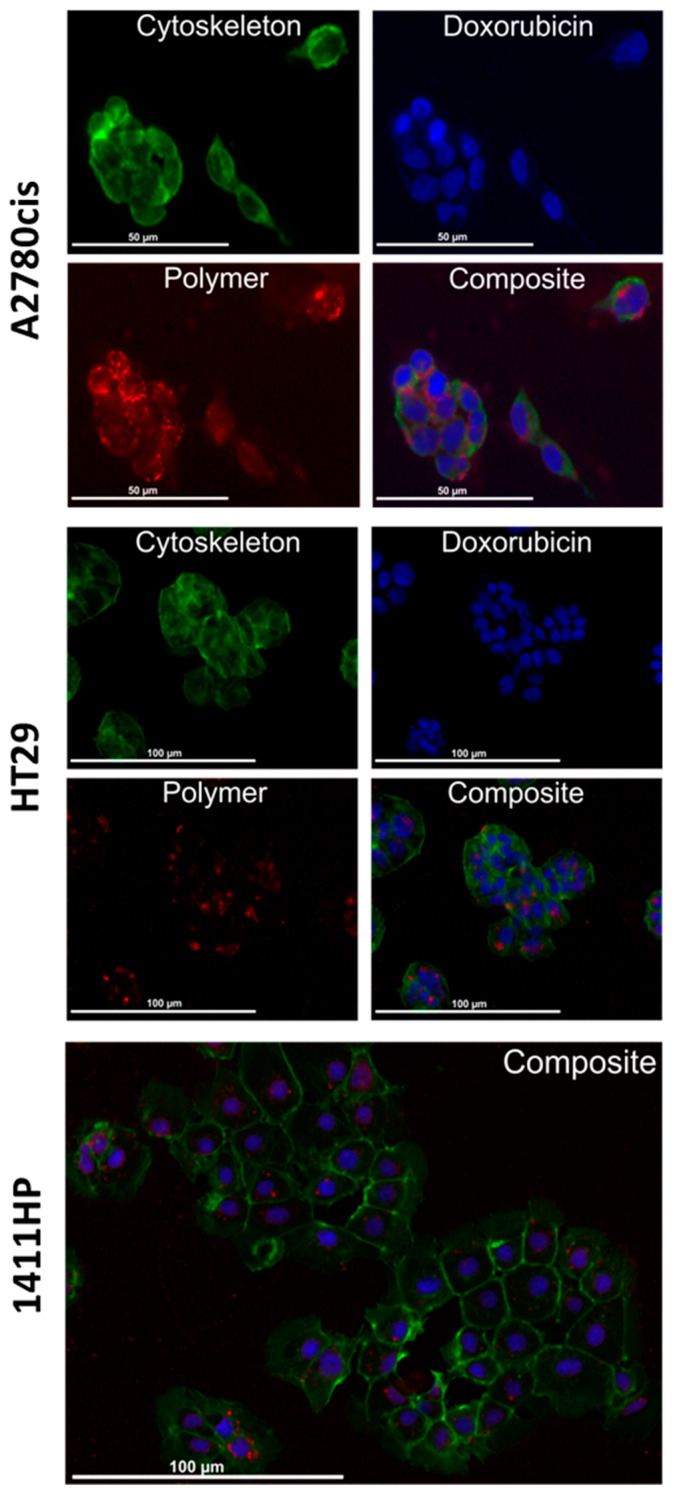
Cellular uptake, distribution and drug release of pHPMA-Dox-Cy7 on cell monolayers. The cells were treated for 8 h and analyzed by multispectral fluorescence imaging, allowing simultaneous visualization of polymer carrier (red), Dox (blue) and cytoskeleton (green) followed by creation of composite images.

Next, we investigated the mechanism of cellular uptake, distribution and drug release of pHPMA-Dox-Cy7, comparing the three cell lines under neutral conditions. As demonstrated by the data shown in Figure 3, inducing cytotoxicity by the conjugate needed a longer exposure time compared to free Dox, which was due to the slower uptake of the whole conjugate. Nevertheless, entering cells as a whole conjugate and thereby circumventing the resistance mechanism underlying the free drug is one major point of the concept of polymer drug conjugates. Therefore, the cells were incubated for 8h and analyzed by multispectral fluorescence microscopy. Although the cell lines exhibited a completely different morphological structure, the distribution pattern of the polymer carrier and Dox was comparable in all three cell lines (Figure 4). A pure Dox-derived signal was visible in the cell nuclei, indicating successful drug release from the polymer backbone and subsequent intercalation in the cellular DNA. The polymer was mainly found in the cytoplasmic area, where it was not homogeneously distributed but confined to subcellular structures and often localized around the nuclear membrane. These findings are in agreement with previous investigations and confirm the assumed mechanism of intracellular drug release from the polymeric carrier. As this cellular mechanism seems to be equal in the three different cell lines, these results corroborated the assumption that rather xenograft tumor specific characteristics are responsible for the different tumor responses in vivo.

### 2.4. Characterization of Cell Line Derived Xenograft Tumors

**Figure 5:**
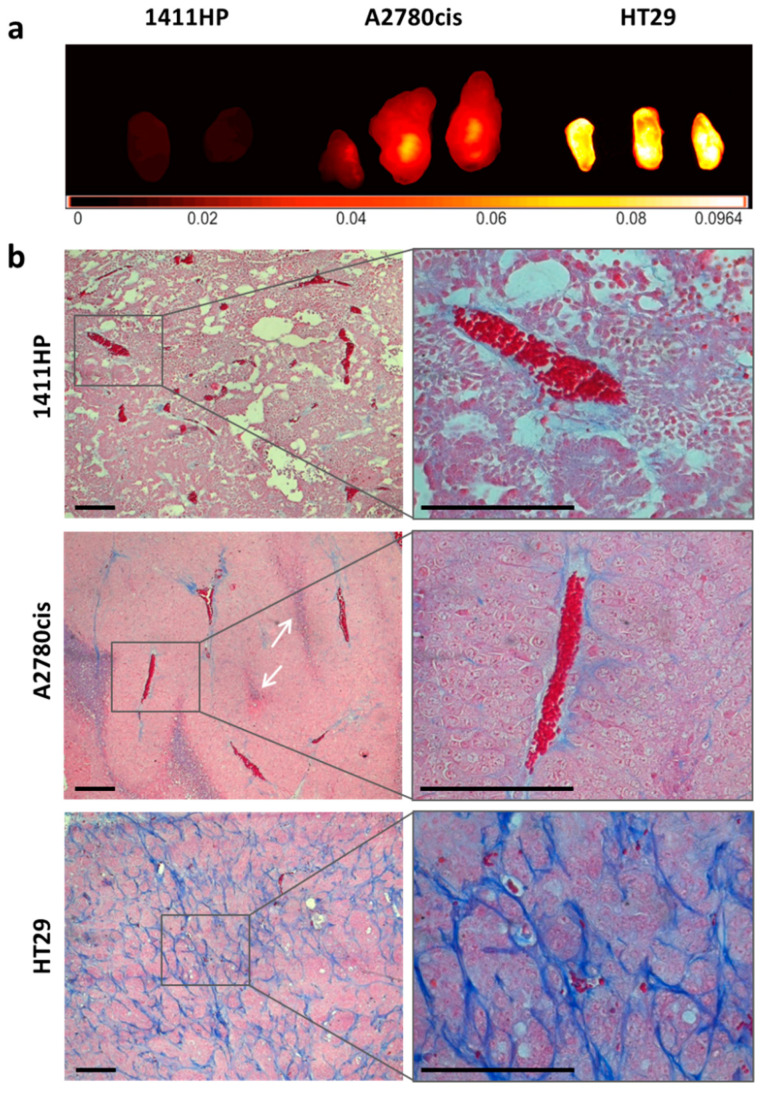
Characteristics of xenograft tumors. (**a**) Ex vivo imaging analysis of sliced tumors 24 h after injection of Hypoxisense^®^ indicating different microenvironments with most hypoxic/acidic conditions in HT29 tumors. (**b**) Azan staining of fixed tumor slides indicating different tumor substructures with large vessels in 1411HP/A2780cis tumors but strongly compacted tumor tissue and occurrence of compressed vessels in A2780cis tumors, whereas HT29 tumors are characterized by small vessels embedded in thick collagen-rich septal structures of abundant fibrotic tissue arranged with tumor tissue. White arrows points to pre-damaged tissue structures featuring A2780cis tumors. (Scale bar: 100 µm).

To analyze the tumor microenvironment of the three different xenograft tumor types, mice were injected with the imaging agent Hypoxisense^®^ (PerkinElmer, Waltham, MA, USA), which is fluorescently labeled and targeted to the carbonic anhydrase IX, indicating a hypoxic/acidic micromilieu. After 24 h, tumors were removed, sliced and analyzed. As shown in Figure 5a, A2780cis tumors were characterized by a more hypoxic/acidic micromilieu compared to 1411HP tumors, which confirmed our previous investigations. Notably, HT29 tumors showed a substantially higher signal compared to A2780cis tumors, indicating a strongly hypoxic/acidic tumor microenvironment. Further characterization was performed by Azan staining, as shown in Figure 5b. Xenografts of 1411HP and A2780cis, as fast-growing tumors, are highly vascularized tumors with large vessels and less extracellular matrix material, whereas the tumor parenchyma is arranged with small septal structures. Notably, A2780cis tumors differ from 1411HP tumors, showing a strongly compacted tumor tissue and occurrence of compressed vessels. A further typical feature of A2780cis tumors is the frequent presence of specific structures comprising injured tissue, with loosely distributed intact erythrocytes which seem to originate from damaged and collapsed vessels (Figure 5b, white arrows). In contrast, HT29 xenografts are rather slow growing, less vascularized tumors with small vessels embedded in thick collagen-rich septal structures, whereas the tumor parenchyma is compacted and is arranged with abundant connective tissue. Together, these analyses revealed clear differences in tumor microenvironment and substructure among the three tumor types.

### 2.5. Intratumoral Distribution and Drug Release of pHPMA-Dox-Cy7 in Xenograft Tumors

**Figure 6:**
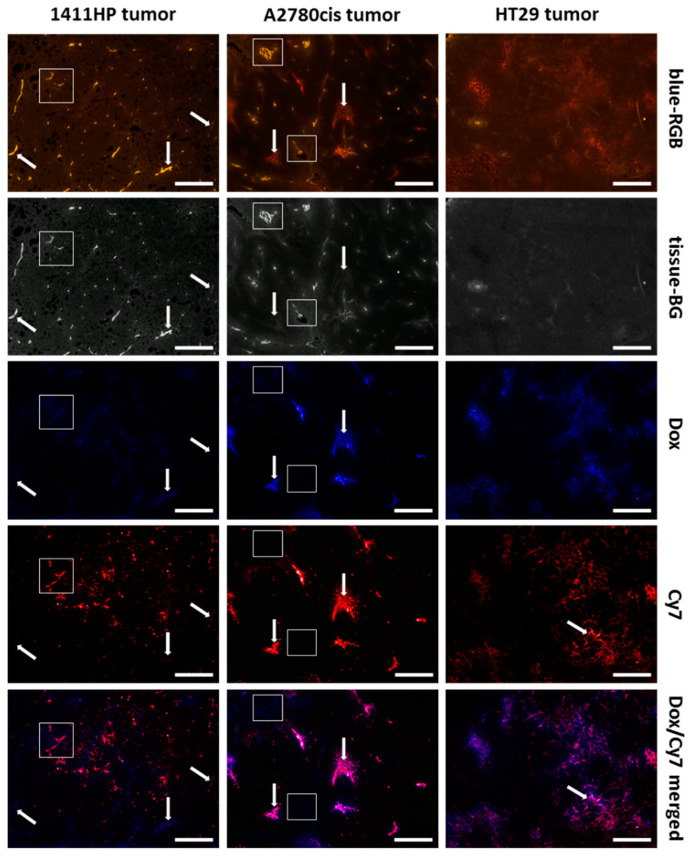
Patterns of intratumoral distribution 24 h after injection of the pHPMA-Dox-Cy7 conjugate. Depicted are images of the same area, respectively, generated under blue fluorescent light (upper row) and after spectral dependent isolation of signals of the tissue background (second row) of Doxorubicin (third row) and Cy7-labeled polymer carrier (fourth row) and as merged version of both (fifth row). In the 1411HP tumor type (left column), combined signals of Dox and polymer carrier are frequently associated with vessel structures (arrows and square). The A2780cis tumor type (middle column) is characterized by intense combined signals of Dox and polymer associated with specific pre-damaged tissue structures (arrows), whereas areas of large intact vessels show pure Dox signals (squares). In the HT29 tumor type (right column), intense polymer-derived signals are confined to the specific septal structures (arrows), whereas Dox-derived signals appear to be in between. (Scale bar: 500 µm).

**Figure 7:**
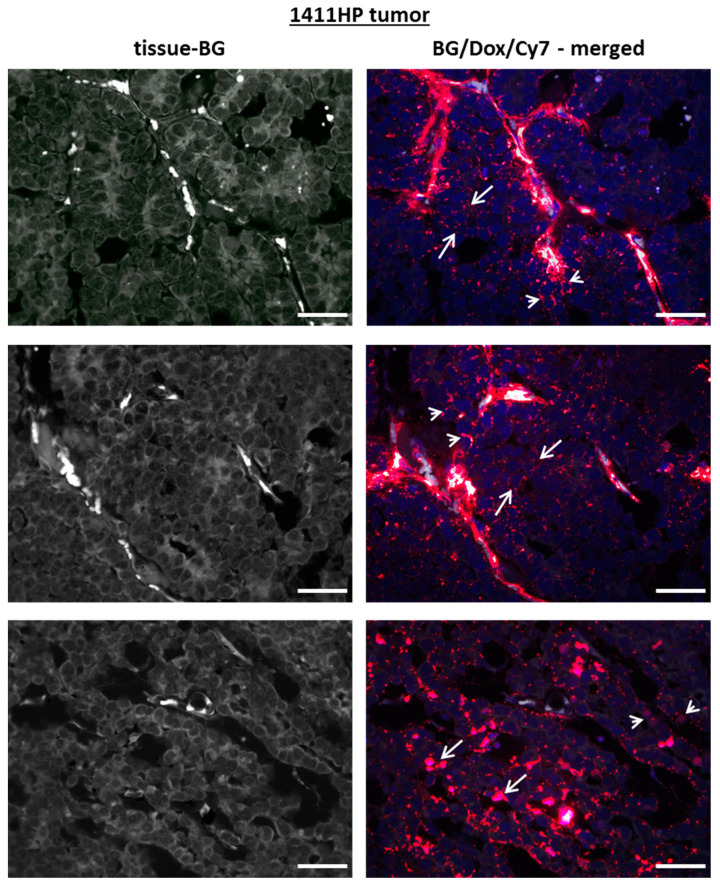
Specific pattern of distribution in the 1411HP tumor type. Depicted are three examples representing the main features of distribution found in the 1411HP tumor type 24 h after injection of the pHPMA-Dox-Cy7 conjugate. Images on the left side show the tissue background, respectively. On the right side, merged images of isolated Dox (blue) and polymer carrier (red) derived signals together with the tissue background are shown, respectively. A substantial amount of the conjugate is still located within vessel structures (upper and middle example) and from there a consistent distribution throughout the tissue seems to occur, eventually resulting in cellular uptake and cleavage reaching the typical pattern of nucleus confined Dox and peripheral polymer (white arrows), including states of cytoplasmic co-localization of Dox and polymer (white arrowheads). Further patterns of the process of distribution can be recognized (bottom example), including the presence of extracellular deposits of uncleaved conjugate (arrow) and nucleus associated combined signals of Dox and polymer (white arrowheads). (Scale bar: 50 µm).

**Figure 8:**
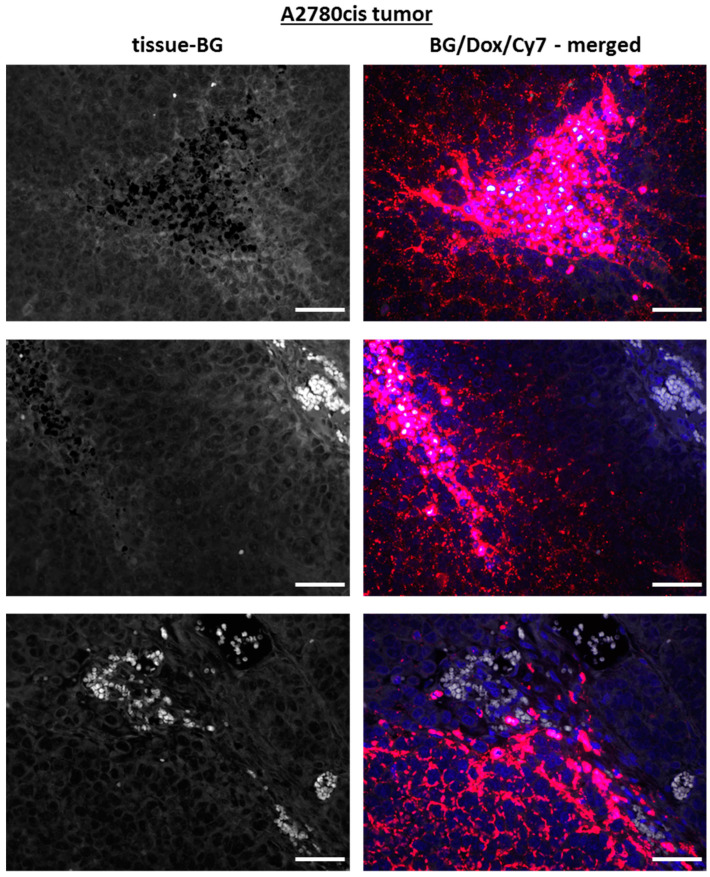
Specific pattern of distribution in the A2780cis tumor type. Depicted are three examples representing the main features of distribution found in the A2780cis tumor type 24 h after injection of the pHPMA-Dox-Cy7 conjugate. Images on the left side show the tissue background, respectively. On the right side, merged images of isolated Dox (blue) and polymer carrier (red) derived signals together with the tissue background are shown, respectively. Concentrated signals of co-localized Dox and polymer indicating the conjugate are confined to specific pre-damaged tissue structures from where the distribution appears to start and proceeds yielding typical patterns of cleavage and distribution in the periphery (upper and middle example). In contrast, areas of large intact vessels are characterized by cells with pure Dox accumulation lacking polymer-derived signals (middle example, upper right corner). Inconsistent distribution and cleavage of the conjugate occurs, resulting in completely different accumulation patterns, i.e., polymer associated or Dox only, within closely connected areas (bottom example). (Scale bar: 50 µm).

**Figure 9:**
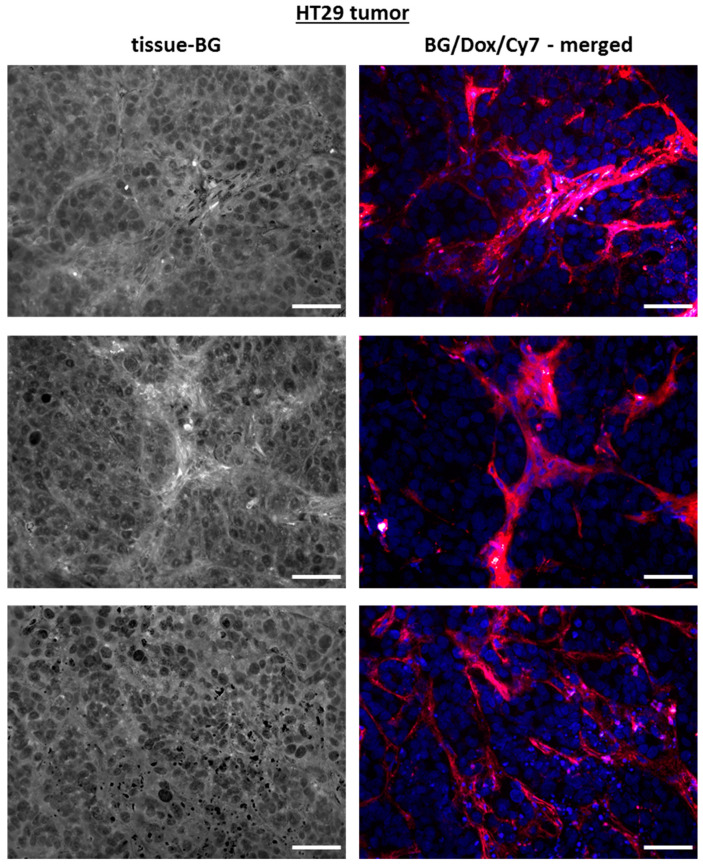
Specific pattern of distribution in the HT29 tumor type. Depicted are three examples representing the main features of distribution found in the HT29 tumor type 24 h after injection of the pHPMA-Dox-Cy7 conjugate. Images on the left side show the tissue background, respectively. On the right side, merged images of isolated Dox (blue) and polymer carrier (red) derived signals are shown, respectively. Intense conjugate- and pure polymer-derived signals are confined to the specific septal connective tissue structures, whereas adjacent tumor cells frequently showed pure Dox accumulation. This suggests a distribution starting from embedded vessels but the entrapment of the polymer and premature release of Dox already within the connective tissue compartment result in the predominant feature of diffusion of pure Dox. Examples with pattern of cytoplasmic polymer can also be found (upper example, bottom right corner). (Scale bar: 50 µm).

Based on the data showing differences in treatment response as well as tumor microenvironment and substructure, we hypothesized that the mechanisms of intratumoral distribution and drug release of the pHPMA-Dox conjugate are also different among the three tumor types. To analyze this, the tumors were removed and fixed 24 h after injection of the conjugate to capture an early phase of the process yet before an obvious tumor response can be noticed. Spectral dependent isolation of true signals by unmixing cubes generated by multispectral fluorescence microscopy enabled simultaneous visualization of both Dox and the Cy7-labeled polymer carrier. First, we generated images with low magnification to capture areas of a few millimeters representing the general characteristics of the whole tumor tissue, respectively. Figure 6 shows a direct comparison of the three different tumor types. The images taken under blue fluorescent light (upper row) were used for orientation. They clearly reflected the typical tumor characteristics in accordance with the data of Azan staining shown in Figure 5 regarding vascularization and tissue density, although the specific thick collagen-rich septal structures of the HT29 tumor type were less visible. The isolated tissue background (second row) similarly differentiated the three tumor tissue types. Notably, a completely different pattern of localization of Dox (third row) and polymer carrier (fourth row) as well as of resulting co-localization of both (fifth row) became visible after isolation of the respective signals and clearly distinguished the three tumor types (Figure 6). Based on these analyses, we generated images with high magnification to elaborate the specific features of each tumor. Examples of each tumor type are shown in Figure 7 (1411HP), 8 (A2780cis) and 9 (HT29), respectively. In the 1411HP tumor type, combined signals of Dox and polymer carrier were frequently observed to be associated with vessel structures indicating the conjugate during circulation before extravasation (Figure 6, left column; Figure 7). From there, a consistent distribution throughout the tissue seems to occur, eventually resulting in cellular uptake and cleavage, reaching the typical pattern of nucleus confined Dox and peripheral polymer. Different stages of the process can be recognized, including the presence of extracellular deposits of the uncleaved conjugate, cytoplasmic co-localization of Dox and polymer, cytoplasmic localization of pure polymer and even nucleus associated combined signals (Figure 7). The A2780cis tumor type was characterized by distinct large areas with intense signals of co-localized Dox and polymer (Figure 6, middle column). These areas could be recognized as those specific structures described above comprising injured tissue with damaged or collapsed vessels. In contrast, large intact vessels containing the uncleaved conjugate could not be found. Instead, the conjugate distribution seems to start from these specific pre-damaged structures and proceeds by yielding the typical patterns of cleavage and distribution in the periphery (Figure 8). Interestingly, these specific pre-damaged structures most likely originate from specific phenomena occurring in solid tumors that have been recently described by Matsumoto et al. as “vascular bursts”, which were associated with enhanced extravasation of nanoparticles [18]. Furthermore, it can be assumed that an early induction of treatment related cell death occurs within these pre-damaged areas. In addition, the presumed hypoxic/acidic microenvironment present in these areas may lead to accelerated cleavage of the conjugate. Notably, areas of large intact vessels were characterized by cells with pure Dox accumulation lacking polymer-derived signals. Moreover, a completely different accumulation pattern, i.e., polymer associated or Dox only, can be observed within closely connected areas, indicating inconsistent distribution and cleavage of the conjugate (Figure 8). The clear differences in comparison to the 1411HP tumor can be explained by the specific features of the A2780cis tumor type. The compressed tumor tissue leads to high interstitial pressure, whereas the co-existence of pre-damaged and intact vessels may result in different pressure gradients. In addition, a premature conjugate cleavage seems to occur in areas of more hypoxic/acidic environment. Together, this suggests a partly hindered distribution as whole conjugate, whereas the released Dox is able to diffuse. The pure Dox signal around large intact vessels hints at possible re-circulation of Dox, confirming our previous assumption. In the HT29 tumor type, intense conjugate- and pure polymer-derived signals were found to be confined to the specific connective tissue structures, whereas adjacent tumor cells frequently showed pure Dox accumulation (Figure 6, right column; Figure 9). This suggests pronounced accumulation of the conjugate in the connective tissue structures directly starting from embedded vessels and premature release of Dox already within this tissue compartment due to the strongly hypoxic/acidic microenvironment. Although this is the predominant feature of the HT29 tumor, examples with a typical pattern of cytoplasmic polymer could also be found (Figure 9). Nevertheless, the polymer appears to be entrapped within the compartment of connective tissue which, in combination with the premature Dox release, prevents distribution as a whole conjugate. The resulting predominant diffusion of pure Dox could explain the similar response pattern observed upon treatment with free Dox and pHPMA-Dox in the HT29 tumor type. Moreover, the preferential accumulation of pure Dox also undermines the concept of using polymer drug conjugates to enter cells as whole conjugate and thereby circumventing the resistance mechanism underlying the free drug. In conclusion, the data suggest different mechanisms of intratumoral distribution and drug release of the pHPMA-Dox conjugate among the three tumor types, which is clearly associated with different tumor substructures and microenvironments.

## 3. Discussion

The aim of the present study was to analyze the intratumoral distribution and drug release of the 200kDa high molecular weight star-like structured pHPMA-Dox conjugate type that we previously investigated, which demonstrated the potential to overcome drug resistance based on high tumor accumulation analyzed by whole tissue fluorescence imaging. However, combined data of analyses performed in three different xenograft tumor models revealed completely different response patterns after treatment with pHPMA-Dox. Xenograft tumors of the cell line 1411HP initially did not respond and showed further growth, which then was followed by a strong response, eventually leading to complete tumor regression. In A2780 tumors, conjugate treatment also induced tumor regression but the onset of response occurred earlier compared with 1411HP tumors and higher treatment-related toxicity was observed. In the HT29 tumor model, treatment with pHPMA-Dox failed to induce regression but rather induced growth retardations and the response pattern did not clearly differ from those observed upon treatment with free Dox. In addition, treatment related toxicities occurred. In contrast to these differences, the in vitro characteristics of the three cell lines in terms of treatment response pattern and mechanism of cellular accumulation and drug release were very similar. Therefore, we supposed that xenograft tumor type specific characteristics such as substructure and microenvironment could be responsible for different mechanisms of distribution and drug release of pHPMA-Dox resulting in the different response patterns. Using a Cy7 labeled variant of pHPMA-Dox and multispectral fluorescence microscopy, we were able to track polymer carrier and Dox simultaneously within the tumor tissues and observed a completely different pattern of distribution in an early phase of accumulation, allowing some conclusions about the mechanisms that may underlie the different responses.

In our previous study, we concluded that the microenvironment of 1411HP tumors initially does not enable efficient drug release, but a time- and treatment-related switch to a more supportive microenvironment occurs, resulting in very effective release of Dox from the highly accumulated conjugate, leading to the specific delayed and strong response. The analyses of our current study confirmed the specific less hypoxic/acidic microenvironment generally present in the 1411HP tumor type but also added new data which require a complementation of initial conclusions. Thus, the process of extravasation of the conjugate from vessels into tumor parenchyma appears to take a longer time in 1411HP tumors compared to the other tumor types since signals of the conjugate were frequently found to be associated with vessel structures. In addition, the less hypoxic/acidic microenvironment prevents premature cleavage, enabling consistent tissue distribution and cellular uptake as whole conjugate followed by intracellular drug release. This eventually leads to the consistent induction of cell death throughout the whole tumor tissue, resulting in strong and complete regression, whereas the microenvironmental switch may be rather a consequence than a cause. Together, this may be considered as the desired mechanism of action of such a therapy approach using polymer drug conjugates with pH-sensitive drug release which, in turn, is only working due to the specific characteristics of the 1411HP tumor, i.e., high degree of vascularization with large vessels, loosely non-compressed tumor parenchyma and less hypoxic/acidic microenvironment.

In the case of A2780cis tumors, it was concluded that the more hypoxic/acidic microenvironment present in this tumor type accelerates the release of Dox from the conjugate, resulting in an earlier response. Moreover, we had speculated that, in part, very early release of Dox immediately after delivery of the conjugate in the tumor tissue may lead to recirculation of a part of Dox, explaining the occurrence of systemic toxicity observed in this model. Interestingly, the analyses of this study support such a notion since we frequently found tissue areas with large intact vessels associated with pure Dox signals. However, due to the complete lack of polymer signal in these areas, this does not support the assumption that premature drug release takes place during or immediately after extravasation of the conjugate from these vessels. Instead, the accumulation appears to start from specific pre-damaged tissue areas comprising injured or collapsed vessels featuring the A2780cis tumor type, a phenomenon recently described by Matsumoto et al. [18]. Subsequently, a similar distribution and cleavage pattern as in the 1411HP tumor seem to occur, but the compressed tumor parenchyma and different pressure conditions may hinder further consistent distribution. In addition, the specific pre-damaged tissue areas probably belong to the most hypoxic/acidic areas within the tumor. Therefore, accelerated release of Dox may take place in parallel, which in turn is able to diffuse throughout the tissue and may also enter intact vessels, resulting in recirculation. Together, this suggests that the specific characteristics of the A2780cis tumor type prevent the desired mechanism of distribution and drug release as postulated for the 1411HP tumor type. Thus, though treatment with the conjugate clearly outperforms free Dox treatment in terms of the therapeutic effect, it does not prevent systemic toxicity. However, the aim of using polymer drug conjugates for treatment is to achieve a higher drug concentration, selective within the tumor tissue, yielding two main effects, i.e., overcoming drug resistance and avoiding the toxicity generally induced by free drug treatment. To accomplish this, the conjugate has to be refined accordingly to meet specific requirements necessary for treatment of tumor types with characteristics represented by the A2780cis tumor model. The high molecular weight and the specific structure of the star-like polymer carrier enable long blood circulation associated with a selective extravasation within the tumor tissue but may also hinder consistent further distribution throughout the whole tumor tissue. Cleavage into low molecular weight units immediately after extravasation of the conjugate could facilitate tissue penetration. The pH-sensitive hydrazone bond between the subunits should be cleavable already at milder acidic conditions, whereas it has to be more stable between the carrier and the drug within the subunits, which enables further distribution as small conjugates, followed by cellular uptake and intracellular drug release. To achieve specific sequential cleavage, the pH sensitivity of both hydrazone bonds has to be adjusted accordingly, which can be accomplished by using specific spacer structures [16]. Together, these improvements could increase intratumoral distribution and decrease occurrence of high amounts of the free drug. In addition, such a conjugate type would also be effective in tumors with less hypoxic/acidic interstitial microenvironments, as represented by the 1411HP tumor model.

In HT29 tumors, the desired mechanism of distribution and drug release mentioned above seem to be almost completely abrogated, which is strongly associated with the specific tumor substructure and the highly hypoxic/acidic microenvironment present in this tumor type. The pronounced premature drug release eventually leads to a response and toxicity profile that do not clearly differ from those of free drug treatment. Thereby, the lack of improved response to treatment with the conjugate could be a result of two different aspects. The primary high accumulation of the conjugate within the connective tissue compartment, which probably directly starts from embedded vessels, may function as a buffer since this tissue component takes a substantial proportion of the whole tumor tissue. In addition, the premature drug release already within this compartment can lead to re-entry of the free drug into embedded vessels and further result in a predominant diffusion of the free drug throughout the tumor tissue, which undermines the concept of this therapy approach. Moreover, the polymer appears to be entrapped in the connective tissue compartment, which may therefore function as a barrier, hindering further distribution. One reason for this phenomenon could be associated simply with the size and structure of the conjugate, but a specific affinity to connective tissue cells or specific chemical interaction of the pHPMA carrier with the collagen-rich matrix featuring this tumor component is also conceivable. Therefore, it is difficult to propose whether a refined conjugate type designed according to the scheme described above would improve the therapy effect in the HT29 tumor type. However, it is worth testing this. On the other hand, if there is indeed a specific affinity of pHPMA conjugates to intratumoral fibrotic structures, it could be utilized for therapy approaches targeting the fibrotic tumor stromal component. Of note, tumor types with a high desmoplastic reaction and a high content of fibrotic stromal tissue, which occur in breast, colorectal and particularly in pancreatic cancer, are considered difficult to treat with conventional systemic therapy but also restrict nanotherapeutic approaches; yet, targeting this tumor component can be a therapeutic strategy [19,20,21,22]. Accordingly, a specific pHPMA conjugate for treatment of such tumor types should contain two different drug components: one directly targeting the fibrotic tissue structures and another targeting tumor parenchyma cells. The resulting degradation of fibrotic tissue structures would decompress the tumor tissue and reduce the interstitial pressure which facilitates further distribution of released small subunit conjugates containing the tumor cell targeting drug. This could improve therapy efficacy in tumor types represented by the HT29 tumor model.

## 4. Conclusions

In conclusion, our analyses show that intratumoral distribution and drug release of and response to pH-sensitive polymer drug conjugates can be completely different and that this phenomenon is strongly associated with the specific tumor substructure and microenvironment, which generally can also be completely different among tumors. As an example, it reflects the pronounced heterogeneity observed in the clinical application of nanomedicines and contributes data explaining the underlying mechanism at a tumor tissue level. Of note, inter-individual patient and tumor heterogeneity resulting in different therapy efficacy and outcomes is generally observed in all approaches to tumor therapy, which requires further development of more individualized therapeutics. Obviously, the same holds true for nanomedicines and several new strategies are under investigation [3,7]. One important aspect of the design of nanotherapeutics is to consider specific characteristics of different tumor types, which in turn can also be exploited for targeted therapy. For this purpose, well characterized preclinical xenograft tumor models that reflect the heterogeneity of patient tumors are useful tools.

## 5. Materials and Methods

### 5.1. Synthesis and Physico-Chemical Characterization of Star-Shaped Polymer Drug Conjugate pHPMA-Dox-Cy7

The semitelechelic linear copolymer of HPMA and *N*-(*tert*-butoxycarbonyl)-*N*’-(6-methacrylamidohexanoyl)hydrazine (MA-Ah-NHNH-Boc) containing amino-reactive thiazolidine-2-thione groups (TT) on the main chain end (polymer 1, Table 1) was synthesized by free radical copolymerization techniques, as described elsewhere [23]. The star precursor (polymer 2, Table 1) was obtained by the controlled grafting onto approach. Polymer 1 was employed for grafting onto PAMAM dendrimer (G2, 16 amino groups), followed by deprotection of hydrazide groups [23].

The pHPMA-Dox-Cy7 conjugate was synthesized by a two-step procedure. First, the fluorescently labeled star copolymer was synthesized by Cy7-NHS-ester attachment to hydrazide groups of polymer 2, thus forming a hydrazide bond, which is stable in a physiological environment. Afterward, the polymer conjugate pHPMA-Dox-Cy7, containing Dox bound via pH-sensitive hydrazone bond, was synthesized using the condensation of hydrazide groups of the Cy7-labeled polymer precursors 2 with keto group of Dox, according to the previously described procedure [24]. The final star polymer was freed of unbound Dox and fluorescence dye using GPC chromatography, using columns filled with Sephadex LH-20 and methanol as eluent.

Molecular weights, *M*_w_ and *M*_n_, and their distribution *Ð* were characterized by the Shimadzu HPLC system containing the photo-diode array, differential refractive index Optilab-rEX and multi-angle light scattering DAWN HELEOS II (Wyatt Technology Co., CA, USA) detectors. The TSKgel G4000SWx (300 × 7.8 mm; 5 μm) column using flow rate 0.5 mL min^−1^ and methanol-sodium acetate buffer (0.3 M; pH 6.5) mixture (80:20 vol.%) as the mobile phase was employed.

The TT group content was determined spectrophotometrically on a Helios α (Thermo Fisher Scientific, Waltham, MA, USA) spectrophotometer (ε_305_ = 10,700 L mol^−1^ cm^−1^) in methanol. The Dox content was estimated similarly (ε_488_ = 9800 L mol^−1^ cm^−1^) in water. The content of the fluorescent dye Cy7 was measured by UV spectrophotometry in methanol, using the molar absorption coefficient ε_750_ = 199,000 L mol^−1^ cm^−1^. The dynamic light scattering (DLS) of aqueous conjugate solutions was measured at the scattering angle of 173° on a Nano-ZS, Model ZEN3600 (Malvern Panalytical, Malvern, UK) Zetasizer. Alternatively, DLS was carried out with argon laser for samples containing the fluorescence dye. The hydrodynamic radius (*R*_h_) was determined by the DTS (Nano) program.

The release of Dox from star conjugate (concentration 0.5 mM Dox) was determined in citric acid-phosphate buffers at pH 5.0, 6.0, 6.5 or 7.4 at 37 °C. The released Dox was analyzed by HPLC, as previously described [25]. All drug-release data are expressed as the amount of free drug relative to the initial drug content in the conjugates. The Dox was used as standard for calibration of the method. All experiments were conducted in triplicate.

### 5.2. Cell Culture, SRB Cytotoxicity Assay, Preparation of Cell Monolayers

Cell lines 1411HP (germ cell tumor), A2780cis (ovarian carcinoma) and HT29 (colorectal carcinoma) were cultivated with RPMI medium (containing 10% fetal bovine serum and 10% penicillin/streptomycin) at 37 °C/5% CO_2_ in a humid atmosphere.

For the sulforhodamine-B (SRB) cytotoxicity assays, cells were seeded in 96-well plates and incubated for 24h. The pHPMA-Dox-Cy7 conjugate was pre-incubated for 24 h in phosphate buffers with different pH values ranging from 5.5 to 7.4. Afterwards, the stock solution (equivalent to a doxorubicin concentration of 1.44 mM) was used directly to prepare serial dilutions (0.001 µM–10 µM) which were added to the cells. After incubation/treatment for 2 h, the supernatant was removed and fresh RPMI medium was added to the cells for another incubation period of 96 h. All following steps were performed according to the SRB assay protocol previously described [13].

For the microscopic examination of fixed monolayer cells, the respective tumor cells were seeded and cultivated in chamber slides. After 24 h, they were incubated with pHPMA-Dox-Cy7 (equivalent to a Dox concentration of 30 nM) for 8 h. Then, the supernatant was removed; the cells were rinsed with PBS and were formalin-fixed. After washing with PBS, the cells were treated with Alexa Fluor^®^ 488 Phalloidin to stain the cytoskeleton. Finally, the cell monolayers were rinsed and preserved using mounting medium to be analyzed using multispectral fluorescence microscopy.

### 5.3. Animal Care and Treatment, Preparation of Tumor Probes and Slides

The animal protocols used in this study were evaluated and approved by the Laboratory Animal Care Committee of Sachsen-Anhalt, Germany (approval code: 203.h-42502-2-1186 MLU). Male athymic nude mice (Hsd:Athymic Nude-*Foxn1*^nu^, male) from the breeding of the ZMG of the Martin Luther University Halle-Wittenberg, Germany, were kept under controlled conditions (12 h day/night cycle, 24 °C). To generate xenograft tumors, mice were short-time anesthetized using isoflurane (Forane^®^, Abbott, Wiesbaden, Germany) and tumor cells suspended in 150 µL of PBS were subcutaneously injected into the right flanks of the mice. Mouse weight and tumor size were measured continuously. Monitoring of tumor growth was performed by caliper measurement and volume calculation using the formula a^2^ × b × π/6, with “a” being the short and “b” the long dimension.

To test the efficacy of the previously studied pHPMA-Dox conjugate [13] in the HT29 colorectal carcinoma model, 5 million HT29 cells were injected subcutaneously per mouse. After establishment of tumors, mice were divided into 3 groups, with 3 mice of equal initial tumor volume per group. Treatment was performed by intraperitoneal injections on days 1 and 4 with 5 mg/kg bodyweight Dox or a 2-fold Dox equivalent dose of pHPMA-Dox (equal to 10 mg/kg bodyweight Dox) or PBS.

For the examination of the tumor micromilieu, xenografts (2 per cell line) were established by subcutaneous inoculation of tumor cells of 1411HP (10 million), A2780cis (10 million) and HT29 (5 million). When tumor volumes reached at least 0.75 cm^3^, the mice received a single Hypoxisense^®^ (PerkinElmer, Waltham, MA, USA) injection (100 µL in PBS; 2 nmol/100 µL). Then, 24 h after Hypoxisense^®^ injection, mice were sacrificed, and tumors were necropsied and cross-sectioned to perform ex vivo multispectral fluorescence imaging using the Maestro™ in vivo imaging system from CRI (Cambridge Research and Instrumentation, Woburn, MA, USA), as previously described [13]. Furthermore, tumor probes were formalin fixed, paraffin embedded and sliced to perform HE and Azan staining according to standard protocols, to be examined with light microscopy.

To study intratumoral accumulation, distribution and cleavage, the fluorescent labeled pHPMA-Dox-Cy7 conjugate was administered as single injection using a 6-fold Dox equivalent dose in 2 mice per xenograft type. After 24 h, mice were sacrificed and tumors were removed, formalin fixed, embedded in paraffin and sliced. Tissue slides from untreated tumors of each xenograft type were used as controls. Prepared tumor slides were dewaxed and rehydrated by decreasing alcohol series from xylene up to bi-distilled water and were embedded in mounting medium (Dako^®^ Fluorescence Mounting Medium, Agilent Technologies, Santa Clara, CA, USA), to be analyzed using multispectral fluorescence microscopy.

### 5.4. Multispectral Fluorescence Microscopy of Fixed Monolayer Cells and Tumor Tissue Slides

For the examination of fixed tumor cells and fixed tumor sections, an upright Leica DM4000B transmitted-light microscope combined with a Nuance^®^ Ex multispectral imaging system from PerkinElmer (Hopkinton, MA, 01748, USA) was used. As light source, a 200 W self-aligning metal-halide lamp (PhotoFluor^®^ II NIR; 360–800 nm) from 89 NORTH™ (Burlington, VT, 05401, USA) was used. Two filter sets containing each a narrow band excitation filter and a longpass emission filter were used for the microscopic examination: blue filter set—excitation filter, 450–490 nm; emission filter, 515 nm longpass; cube acquisition from 520–720 nm (10 nm steps); near infrared (NIR) filter set—excitation filter, 710–775 nm; emission filter, 780 nm longpass; cube acquisition from 785–950 nm (10 nm steps). For the cube acquisition, an automatic exposure tool was used to avoid over- or underexposure. Nuance™ 3.0.2 software (PerkinElmer) was used to evaluate the acquired cubes, which consisted of a series of images taken at specific wavelengths containing the spectral information of the whole wavelength range. The obtained single spectral species of doxorubicin, of Alexa Fluor^®^ 488 Phalloidin, which was used to stain the actin cytoskeleton of the cells, and of the NIR-dye Cy7, which served as the polymer label, were separated from background and autofluorescence spectra using the Nuance™ software (version: 3.0.2.). In the case of tissue slides, specific background spectra were generated using untreated xenograft tumor samples. The images of the single spectral species were colored differently and used to create composite images.

## Figures and Tables

**Table 1:** Characteristics of polymer precursors and polymer 3, designated as pHPMA-Dox-Cy7 conjugate.

Polymer	*M*_w_	*Ð*	*R*_h_ (nm)	Hydrazide Content (mol.%)	Number of Arms ^b^	Dox (wt.%)	Cy7 (wt.%)
1 ^a^	23,700	1.6	3.0	-	n.a.	-	-
2 ^a^	165,000	1.7	9.4	5.2	7	-	-
3	~180,000 ^c^	~1.8 ^c^	10.8	-	7	10.6	0.76

^a^ Star-like dendrimer-based polymer precursor. ^b^ Number of polymer arms connected to the dendrimer core, calculated as ratio of molecular weights of star and linear precursor and from remaining amino groups on dendrimer after grafting reaction. ^c^ Estimated *M*_w,_ *Ð* and *R*_h_ from GPC analysis.

## Abbreviations

MDPI	Multidisciplinary Digital Publishing Institute
DOAJ	Directory of Open Access Journals
TLA	Three letter acronym
LD	Linear dichroism
